# Milksickness

**Published:** 1853-08

**Authors:** Samuel Thompson


THE NORTH-WESTERN

MEDICAL AND SURGICAL JOURNAL.

NEW SERIES.

VOL II. AUGUST, 1853. No. 4.

### ORIGINAL COMMUNICATIONS.

ART. I.—*Milksickness*; by SAMUEL THOMPSON, M. D. Read before the Æsculapian Society, Lawrenceville, May 1853.

I MUST request from the society a lenient judgment on this paper, as till a few days back, various circumstances had driven from my memory the fact, that I was appointed to write a paper on this subject.

Much has already been written on the cause of this disease, nevertheless, I believe, we are yet very far from being unanimous in our views. Indeed, all reasoning thereon which has hitherto been presented, appears to have been, either upon analogy, upon popular belief, or, worse still, upon the assumption, that because one fact followed another assumed fact, the sequence was a consequence. Perhaps, in the present stage of our knowledge, we shall not gain much by discussions on the remote cause of this disease; at any rate, in this paper, I shall not spend much time upon it. The pathological conditions have a more immediate bearing upon our practice, and to them I shall therefore chiefly direct my remarks. Symptoms are the signs of disease; by them we classify and define. What, then, are the symptoms or distinctive signs by which we shall know this affection? It is undoubtedly true, that most diseases give rise to effects, which we call symptoms, only *after* such diseases have been in operation some time, or so long, at least, as to have produced derangement of one or more of the functions of health; but it would appear, that the time during which the disease, called milksicknes, is in a state of nascency, is longer than that of most diseases, or, at least, longer than what has, as yet, been perceived, in reference to the majority of the ailments to which the human frame is subject. According to popular belief, a man may suffer from tires, or slows, as they are commonly called, for weeks, and even months, and sometimes the threatening disease is never developed, or, in other words, the powers of nature, either by themselves, or by the aid of medicinal stimulus, remove the cause, and the patient recovers his previous health. These *tires* constitute, then, the first symptoms of the disease. But it will be well to define what is meant by the term *tires.* As I have repeatedly heard it explained, it consists in a gradually increasing lassitude, an inability for exertion, a sensation as if the feet and legs were loaded, or the will had lost its power to give energetic motion to them; after a time any muscular efforts give rise to dyspnœa, and sometimes to severe palpitation of the heart. The skin is at times profusely moist, at other times dry and disposed to be cool. The appetite is capricious, occasionally there is nausea. The intellect feels dull. The shades and various combinations of these symptoms, of course, differ in different individuals and in different attacks, even in the same individual; sometimes these symptoms are noticed for only a few days, at other times for weeks, months, and even for more than one season, before the distinctive features of the disease show themselves. These last are often brought on suddenly by some unusual effort, especially if made on a warm sultry day; then, perhaps, the patient suddenly feels himself unable to accomplish what he has begun. Vomiting occurs; as a general rule, the alvine discharges, which, before this, have been for some time irregular in quantity and quality, are now pretty much suspended; in some cases the most obstinate constipation exists. During the first day or two, there is *generally* a little heat of skin, but it rarely lasts longer than this, and during the rest of the attack the integuments are rather dry and cool (not cold). The pulse varying, perhaps, from 80 to 100, rarely exceeding this, and in most cases continuing, from day to day at the same number of beats; its impulse rather slow and laboring, but always very excitable, so that a judgment, based upon the condition of the pulse when you first went to the bedside, or after the patient had been talking, or vomiting, or using any exertion whatever, would sadly mislead the observer. The breath is in most cases horribly fœted, the tongue moist but furred a little down the centre. There is mostly some head-ache, whilst in a large majority of cases, sooner or later, the pupils of the eyes become dilated and a little amenable to the stimulus of light. The hand placed on the abdomen, will feel the aorta heavily pulsating, an indescribable despondency and misery seems to weigh on mind and body, the appetite is gone, while the distress is aggravated by a sensation of burning in the stomach and œsophagus, with intense thirst and craving for cold water, which, with food or medicine, is immediately rejected. The ejected matter, being in most cases green or resembling indigo water, generally exhibiting a distinctly acid reaction with any alkali. In some cases the matters vomited are like frog spawn in spring, and the long strings of tenacious mucus, adhering to the fauces and tongue, greatly increases the distress of the patient.

These, then, are the symptoms of the disease. Symptoms, let us bear in mind, are *signs* of effects produced. What are the effects, then, to which those symptoms point? The long premonition, and the very gradual progress of these effects, are, I think, unmistakable testimony, that the cause, be it what it may, whether vegetable or animal, atmospheric or animalcular, is one of but little potency; that it is one against which the functions of life hold long and sometimes successful struggle; that the obtuseness of the sensations, the offensiveness of the breath and of the excretions, when obtained, are clear manifestations that the cause acts as a depressing or paralyzing poison, gradually suspending the eliminations from the body of those effete materials, which become poisons when retained; and from this same condition may also be understood, the laboring pulse, the abdominal pulsations, the dilated pupils, the cool dry skin, the intestinal torpor. The excitability of the organic nerves is obtunded, the heart is unable completely to expel the blood from the ventricles, and thus the abdominal aorta is no longer *filled*, the blood flows in separate waves, giving rise to the feeling of pulsation. The sensibility of the retina to light, is so far decreased, that the iris hardly contracts when the eye is exposed to a bright light. The skin is cool, evidently showing that vital heat is not wholly, (at least), dependent on combustion; for the pulse, we see, is above the average of health, while the cutanous temperature is below the normal standard.

The intestines appear to have lost their peculiar sensibility, and no longer feel the stimulus of the fæcal mass; consequently its progress is retarded or suspended. The liver, to a great extent, suspends its functions, the portal circle is obstructed, the imperfectly evacuated ventricles refuse to receive the blood in its circuit, the veins of the stomach are therefore retarded in their currents, and, associated with the nervous derangement, produce oppression, nausea and heartburn; the latter, perhaps, in some degree dependent on the morbid secretion of acid by the stomach, at a time when even the ordinary call for such in the function of digestion is suspended. Day by day the evil increases; for, day by day the accumulation of effete matters is greater. The blood is more impure, the nervous centres are more deeply impressed. It appears to me, indeed, that the disease in question, is one of arrested excretory function in the first stage; of excrementitious poisoning in the second; that its effects are so much the greater, the prostration just so much the more severe, because the cause was so slow in its action, so gradual in its effects, the undermining of the bulwarks of health so insidious. I assume, then, that it is a disorder of function, not a disease of structure, and what few post-mortem examinations I have heard of, justify this view; for, except a congested and irritated appearance of the stomach, &c., a natural consequence of the constant straining to vomit, no pathological changes have been noticed. How far does the *treatment* usually employed sustain this view? Every practitioner, be his theory of its cause what it may, trusts, in a great measure, to purgatives If he can remove from the body the foul and fœtid intestinal contents, and can obtain from the internal lining membrane a free secretion of what we know must be abstracted from the blood, he trusts his patient will recover, and generally he is justified in his prognosis. Whenever the skin becomes moist he feels his hopes are high; for natural diaphoresis, during the reign of the morbid cause, is what, I believe, no one ever saw. When the mouth begins to manifest the effects of mercury, he says, my patient will get well;—as many used to believe, as some still believe, *because* the *ptyalism is* a *cure*, the *discharge* salutary. But *really*, *I* am satisfied, it is *only because* the mercury—the effects of which we can *see* in the *mouth*, to which we can look as a thermometer—is then beginning to produce its specific or peculiar action upon all the mucus surfaces, restoring tone to the capillary circulation, and thereby restoring capillary function, that is secretion, and by the aid of secretion the causes of the disease will be removed.

It is a singular fact, that throughout this disease, the renal secretion *appears* little affected, in quantity, at least; whether this is really so, as regards its constituents, I have not ascertained, though the urine *looks* dull and oily. But this, I know, that attention to *stimulating* the renal secretion, has always, in my hands, appeared to be an important aid towards a removal of the disease; but whether this so appeared, only because the normal condition of the urine was thereby restored, or in virtue of a vicarious service performed by the kidneys, I am not prepared to say; the last, however, has been the object sought by me, and this again would tend to show, that by whatever excretory function you can depurate the blood, you in this disease, at least, aid nature to return to the normal performance of her functions. But while this is the end, which, it appears, all ought to seek, for the reasons I have stated, and towards which all the remedies usually employed have a tendency, there is, as we all know, a peculiarity about this disease, which often renders the use of such means futile, the attainment of this end peculiarly difficult. We desire to act upon the intestinal canal by the way which seems most natural, and is most common, and by agents, which experience has taught us, will in other cases, generally produce the desired effects; but in this case, the stomach, that gate-keeper of the bowels, refuses to entertain our agents, and hence, an amount of morbid cause, which, it is probable, might under other circumstances be easily removed, seems hardly amenable to any appliance we can use; when, in fact, from the congested condition of the stomach, and the general sluggish functional actions, *larger* doses than ordinary are needed. Doses *smaller* than usually required, can hardly be retained, and the congested condition of the gastric mucus membrane, impeding absorption, will, I believe, explain the harmlessness of the enormous doses of calomel, which have sometimes been administered, and for which, as in similar treatment of cholera, it has been assumed that they were useful, because they did no harm. Another difficulty attending this morbid irritability, is, that it *appears* needful to use opium or morphia to allay it, and which, when used, must necessarily, to some extent, at least, unless the disease be spasmodic, still further add to the peristaltic and secretory torpor. Then, it will be asked, how is this irritability of the stomach, which so sadly interferes with the exhibition of our medicines, to be remedied, and how are the suspended functions of the bowels to be restored? These are, indeed, important questions; for, to move the bowels and to restore the secretory and excretory functions, are, indeed, our primary indications. But this is not enough, we have for the time to stimulate them to *extra* efforts; for they must not alone do each hour's accruing duty, they must atone for past deficiencies, they must sweep out the poisonous accumulations under which the powers of life are crushed. To restore these functions, and thus to stimulate them to unusual action, what means have we at command? Calomel is supposed to possess this power, and, wisely and moderately used, I believe, it will not disappoint us for some of our purposes. The difficulty to which I have pointed, namely that in the condition of the stomach present in this disease, *absorption*, (the only mode in which I believe calomel acts) is slow and uncertain, and it even, is apt to be rejected when given in large quantities. My practice is generally, to give only about two doses of about ten grains each, rubbed up with an equal quantity of fine *dry* salt. This I place *dry* on the root of the tongue, and immediately give a *small* drink of cold water to wash it down with, and give the other dose in about four hours. I then trust to other means, for a time, at least. I never knew calomel, so given, to be rejected, and, aided by injections, it will rarely fail in about 10 or 12 hours to bring away feculent fœtid stools, generally black and like thick tar, and so horrible offensive, that the odor is almost insupportable. Where the stomach will bear it, I then use strong infusion of senna and salts in small and frequently repeated doses, or cremor tartar and jalap; our more potent cathartics, such as croton oil, gamboge, &c., being from their nauseating effects liable to agravate our most unmanageable symptoms, at least, such has been my experience. In very obstinate cases, where injections with a common syringe do not succed, an enema pump with an œsophagus tube attached, which should, after well greasing, be introduced into the rectum nine or ten inches, so as to pass beyond the annulus of O'Bierne, I have never yet known to fail in relieving, for the time, the constipation. And we should never forget that the retention of such fœtid matters is still further adding to the nervous prostration. I am generally willing to allow my patients good ale, the newer the better, as it not only isa narcotic stimulant, but a laxative. In most cases I have been in the habit of giving a pill containing quinine gr. ijss. and morphia gr. 1-10th ter die, and I think with advantage, to relieve the nausea, to neutralize the acids in the stomach, to supply materials to restore the blood to a normal condition, I give soda bicarb. about ℨss., or, which I generally prefer, Dr. Steven's saline powder, ℨss. in a cupfull of water every two or three hours. Where the breath and stools are very fœtid a few drops of chloride of soda in water, is often useful, and in the latter stages of the disease I think I have found benefit in the following pills, giving one every 3 or 3 hours :

| | | |
|---|---|---|
| Alcoholic Ext. Nux Vomica | ℈j | In Pills xl. |
| Rhubarb . . . . . . . . . . . . . | ℨss. | |
| Aloes Socot. . . . . . . . . . . | ℨss. | |

adding calomel grj. to each pill if indicated (see Brathwaite's Retrospect No. 18, page 116). This I use with the object of restoring tone to the intestinal muscular fibres. I have thought the addition of powdered capsicum might be advantageous in this stage of the disease. It must be evident from the views I have advanced as to the nature of this disease that it can never be considered to be really removed until all the organs of secretion and excretion are fully restored to their proper tone and functions ; so that their products are perfectly free and normal. Unfortunately our patients usually get tired of our attendance before this state of matters is established ; and hence has arisen in some part the general impression of the proneness to relapse, in those who have suffered from milksickness, and the popular idea that those who have once suffered from it are never afterwards quite well. Another cause undoubtedly is, that in most cases those who have been attacked, still continue to reside in the same localities.

But there are circumstances connected with this disease, which though perhaps applying to all diseases, have not, I think, been recognized as belonging to milksickness, viz: that it is not only a disease of long inception, but that it is one varying in its degrees of virulence in different places and in different years, that in fact it is subject to the modifications of epidemic causes.

It has struck me, and I wish that others would state whether it has occurred to them, that ever since the cholera has been naturalized amongst us, its peculiar diathesis, if I may use the phrase, has accompanied not only our fevers, commonly so called, but that the disease in question has become much affected by it, in some respects mitigating its virulence, but in other ways complicating its treatment.

Of these modifications of the disease in question, the more ready amenability of the bowels to the action of purgatives, is the most prominent, and is certainly in one respect an advantage, but inasmuch as it is accompanied by an incapability of bearing the amount of purging, which appears necessary to the complete removal of the causes of the disease, it also becomes a serious and unfavorable modification. I have recently seen several cases of sickness (one especially) bearing all the features of this disease, in which as soon as even moderately free action of the bowels was obtained, a dysenteric character was manifested in the stools; to combat this, as there were signs of ptyalism, I gave spirits of turpentine in doses of a tea-spoonful with four or five drops of laudanum every three hours, and continuing the use of enematæ, with the compound pills before named, the cases all went on to a favorable termination. I have neither time nor space to copy cases. Of the subjects of locality and etiology, I have before recorded my views; but in the absence of well classified facts upon which to base our theories, and as sometimes, what may claim no higher rank than speculations, may lead other minds on fresh lines of investigation. I will mention an idea which occurred to me during the unusual prevalence of the disease called milksick among the cattle in my neighborhood, during the past autumn. It is well known that the leaves of plants and trees produce great effects upon the atmosphere, that during the day time they absorb carbonic acid gas, and give out oxygen, and that during the night a reverse process is going on. Now, as far as my recollection serves me, true milk sickness is most prevalent in very dry seasons, and very rarely occurs till the leaves begin to fade, (a condition occurring earlier in dry seasons); and therefore cease to perform this function of absorbing carbonic acid gas, and I believe it is allowed that all vegetable leaves perform this office. Assuming, therefore, that at the time they cease to execute this duty, that the same amount of carbonic acid gas is evolved as during the period of their absorptive function, especially when the early failure of the leaves would lead to the idea, that from the continuance of hot weather this would be the case, while from the absence of rains, there would be no water to absorb this excess, and it would thence seem probable that there should be an excess of carbonic acid floating in the atmosphere. I give these but as crude ideas, by no means assuming that carbonic acid gas is the sole cause of the disease, but thinking it possible that in some mode, or combination, it may act as a depressing influence, thus giving potency of effect to other causes, or perhaps gradually inducing that functional torpor which I believe to be the essential nature of the disease.

As somewhat corroborative of the opinion that a dry season is conducive to the development of milksickness, I will quote from a most sensible article by Professor L. P. Yandell, on milksickness, published in the *Western Journal of Medicine and Surgery* for May, 1852, but which I had not the pleasure of seeing till the foregoing paper was written, the opinions of various writers as to the season of the year and the character of the seasons, when this disease generally appears. Dr. Smydth says: "The season at which milksickness most prevails is the autumn, after vegetation has been killed by the frost, and has been observed to be most prevalent during dry seasons, when the streams are very low."

Dr. Armstrong says: "That the disease is more prevalent during a dry than a damp season."

Dr. Senton says: "That in wet or rainy weather they had few cases of milksickness, either in persons or stock;" "it was always arrested by one good sweeping rain;" "that in Indiana it is always feared in dry weather."

Dr. Drake remarks: "That on the Miami River it oftenest occurs in August, and continues till the approach of winter, when it generally, but not always, subsides."

Dr. Thompson (Transylvanian Journal of Med.) says: "The season of the year at which the disease is most prevalent is the fall, after vegetation has been mostly killed, &c."

Dr. White says: "The disease is most severe during the dryest season of the year, &c."

Dr. M. L. Dixon says: "The affection is most prevalent from May to November,—but he adds, during very mild seasons, when vegetables continue verdant through the autumnal months, the case may be very different."

I quote all these from Dr. Yandell's very excellent article to shew that I am not quite alone in my idea of the kind of weather when it is most prevalent nor in the condition of vegetation at the time, not that these gentlemen drew the same conclusions as I here suggest.

Dr. Banks, of Charleston, Ill., as quoted in the Rep. on Practical Medicine, of the Illinois State Medical Society, for 1851, remarks: "So far as my observations go, it is worse in dry seasons, but not the dryest seasons."

As to whether my hypothesis points to a truth, as to whether the cause of the coincidence in which so many agree, is what I have suggested, I do not pretend to say. I have offered it but as a hypothesis, an idea sent off as a feeler for truth, not as an opinion formed. In such capacity an offspring of the imagination even may legitimately become a guide to truth.

